# Satellite-derived estimations of spatial and seasonal variation in tropospheric carbon dioxide mass over China

**DOI:** 10.1002/ece3.823

**Published:** 2013-10-03

**Authors:** Yuyue Xu, Changqing Ke, Juanle Wang, Jiulin Sun, Yang Liu, Warwick Harris, Cheng Kou

**Affiliations:** 1 Jiangsu Provincial Key Laboratory of Geographic Information Science and Technology, Nanjing UniversityNanjing, 210093, Jiangsu Province, China; 2 State Key Laboratory of Resources and Environmental Information System, Institute of Geographic Sciences and Natural Resources Research, Chinese Academy of SciencesBeijing, 100101, China; 3 Department of Chemistry, Nanjing UniversityNanjing, 210093, Jiangsu Province, China; 4 Landcare Research, Crown Research InstituteLincoln, 7640, New Zealand

**Keywords:** Carbon dioxide mass, China, emission estimates, satellite sensing, troposphere

## Abstract

China has frequently been questioned about the data transparency and accuracy of its energy and emission statistics. Satellite-derived remote sensing data potentially provide a useful tool to study the variation in carbon dioxide (CO_2_) mass over areas of the earth's surface. In this study, Greenhouse gases Observing SATellite (GOSAT) tropospheric CO_2_ concentration data and NCEP/NCAR reanalysis tropopause data were integrated to obtain estimates of tropospheric CO_2_ mass variations over the surface of China. These variations were mapped to show seasonal and spatial patterns with reference to China's provincial areas. The estimates of provincial tropospheric CO_2_ were related to statistical estimates of CO_2_ emissions for the provinces and considered with reference to provincial populations and gross regional products (GRP). Tropospheric CO_2_ masses for the Chinese provinces ranged from 53 ± 1 to 14,470 ± 63 million tonnes were greater for western than for eastern provinces and were primarily a function of provincial land area. Adjusted for land area troposphere CO_2_ mass was higher for eastern and southern provinces than for western and northern provinces. Tropospheric CO_2_ mass over China varied with season being highest in July and August and lowest in January and February. The average annual emission from provincial energy statistics of CO_2_ by China was estimated as 10.3% of the average mass of CO_2_ in the troposphere over China. The relationship between statistical emissions relative to tropospheric CO_2_ mass was higher than 20% for developed coastal provinces of China, with Shanghai, Tianjin, and Beijing having exceptionally high percentages. The percentages were generally lower than 10% for western inland provinces. Provincial estimates of emissions of CO_2_ were significantly positively related to provincial populations and gross regional products (GRP) when the values for the provincial municipalities Shanghai, Tianjin, and Beijing were excluded from the linear regressions. An increase in provincial GRP per person was related to a curvilinear increase in CO_2_ emissions, this being particularly marked for Beijing, Tianjin, and especially Shanghai. The absence of detection of specific elevation of CO_2_ mass in the troposphere above these municipalities may relate to the rapid mixing and dispersal of CO_2_ emissions or the proportion of the depth of the troposphere sensed by GOSAT.

## Introduction

Carbon in the atmosphere exists in two main forms: carbon dioxide (CO_2_) and methane (CH_4_). CO_2_ is the more important greenhouse gas of the two although CH_4_ produces a larger greenhouse effect per volume than CO_2_, and it exists in much lower concentrations and is shorter-lived than CO_2_ (Denman and Brasseur 2007). In the last two centuries, human activities have considerably altered the global carbon cycle, and most significantly, the mass of CO_2_ in the atmosphere. CO_2_ emissions in China increased by 17 million tonnes, from 1990 to 2003, an increase of 73%, making China the world's second largest carbon emitter after USA during this period (Zou et al. 2009; Guan et al. 2012). According to estimates by late 2006, China overtook USA as the greatest CO_2_ emitter in the world (Gregg et al. 2008; Gurney 2009). In 2010, near to 25% of global CO_2_ emissions from energy systems and industrial processes originated in China (Marland 2012). China, the most populous country of the world, is now well within the 6- to 19-tonnes/person range spanned by the major industrialized countries (Olivier et al. 2012). Carbon emissions from energy consumption in China increased more than 148% from 1997 to 2009, but the spatial pattern of high and low emission regions in the country did not change greatly (Chuai et al. 2012). China, because of its large population, diverse environments, and rapid rate of industrial development in recent years, is a critical location and geographical entity for research on global biological changes. Therefore, accurate estimation of spatial and temporal tropospheric CO_2_ mass change over China is of great importance in furthering our understanding of their effect on the carbon cycle and indispensable to scientific study or policy actions aiming at prediction and control of the climate change (Berezin et al. 2013).

In order to provide baseline references for the reduction in greenhouse gas from China, the statistics CO_2_ emissions from energy use and their impact on the environment are an important aspect to consider. Li et al. (2010) discussed the correlation between carbon emissions and the influencing factors in China from 1953 to 2006 and found that economic growth, population growth, and the evolution of industrial structure had acted to steadily increase China's carbon emissions. Further, Xie et al.(2011) and Zhao et al.(2011) found that foreign direct outward investment or direct investment can reduce carbon emission. Using the grey correlation model, Lin et al. (2007) examined the relationship between CO_2_ emissions and economic development and analyzed energy consumption in 37 departments of Taiwan. Their results indicated that economic growth was a major factor affecting carbon emissions.

Many studies based on statistical data have focused on China's CO_2_ emissions (Streets et al. 2001; Guan et al. 2008; Peters et al. 2012). However, China has frequently been questioned about the data transparency and accuracy of its energy and emission statistics (Guan et al. 2012). Compared with ground-based data statistical methods, satellite remote sensing now offers an effective way to continually, rapidly, and dynamically monitor large-scale CO_2_ distributions (Wiens et al. 2009). In recent years, space-borne remote sensing systems that provide large spatial and temporal coverage have been employed for the measurement of CO_2_. These systems include the SCanning Imaging Absorption spectroMeter for Atmospheric CHartographY (SCIAMACHY) (Bovensmann et al. 1999), Tropospheric Emission Spectrometer (TES), the Infrared Atmospheric Sounding Interferometer (IASI), and the Atmospheric Infrared Sounder (AIRS) (Chahine et al. 2008; Xiong et al. 2008; Xu et al. 2012). Compared with these systems, the Japanese Greenhouse Gases Observing Satellite (GOSAT), launched on 23 January 2009, was the first satellite specifically dedicated to the measurement of greenhouse gases. The NASA's Orbiting Carbon Observatory 2 (OCO-2), expected to be launched in 2015, will further improve the capacity to monitor greenhouse gases (Sakuma et al. 2010). Berezin et al. (2013) estimated multiannual changes of CO_2_ emissions in China using multiannual satellite measurements of tropospheric NO_2_ columns. There have been several studies of tropospheric CO_2_ concentration using GOSAT data (Gisi et al. 2012; Hammerling et al. 2012), but there remains the opportunity for further applications of the data for specific purposes, and in the case of our study, its application to the area of China.

As the first objective of this study, we estimated the tropospheric CO_2_ masses over provincial areas of China during 2010 based on data obtained from GOSAT. The spatial and temporal distributions of tropospheric CO_2_ mass over China were then mapped and discussed and the uncertainties of our results calculated. A further objective was to analyze relationships between our estimates of tropospheric CO_2_ mass and provincial statistics of CO_2_ emissions from energy use. The last objective was to examine relationships between these estimates and statistics with provincial human population numbers and gross regional product (GRP).

## Data Sources and Methods

### Carbon dioxide concentration data

Greenhouse Gases Observing Satellite, launched successfully on 23 January 2009, was the first spacecraft to measure atmospheric concentrations of CO_2_ and CH_4_ from space (Saitoh et al. 2009). GOSAT flies at an altitude of approximately 666 km, completes one revolution of the Earth in about 100 min, and returns to the same point in space in 3 days. The observation instrument on-board GOSAT, the Thermal And Near-infrared Sensor for carbon Observation (TANSO), is composed of two subunits: the Fourier transform spectrometer (FTS) and the Cloud and Aerosol Imager (CAI) (Naoko et al. 2009).

The FTS and CAI data that the satellite collects are first received and processed at JAXA Tsukuba Space Center, Japan. Then, these data are transferred to GOSAT DHF via Tsukuba WAN, a high-speed wide area network in Tsukuba. GOSAT DHF gathers reference data, such as meteorological data, necessary for the higher-level data processing, from cooperating institutions on a regular basis (Watanabe et al. 2010). Among all spectra obtained with FTS, only those measured under no cloud interference within field of view (FOV) are selected for further processing. This screening uses the images from CAI. Based on the absorption characteristics of the gases, the selected spectra are analyzed, using a numerical calculation scheme called the retrieval method, to calculate column abundances of CO_2_ and CH_4_ (Yoshida et al. 2011; Berezin et al. 2013)_._

Changes in CO_2_ concentration are most obvious near the surface of the earth. The CO_2_ absorption bands near 1.6 μm and 2.0 μm are important as absorptions in these bands provide information on the near-surface concentrations. The absorption band around 14 μm is used for obtaining information mainly at altitudes above 2 km (Yoshida et al. 2011).

The FTS SWIR Level 3 data products are generated by interpolating, extrapolating, and smoothing the FTS SWIR Level 2 column-averaged mixing ratios of CO_2_ and CH_4_ on a monthly basis. A geostatistical calculation technique, called kriging, is applied. The values are gridded to 2.5-degree cells. Standard error stored in the Level 3 products is the square root of the estimated average of square errors, that is, between 1 and 2 parts per million (ppm) for most pixels. The FTS SWIR Level 3 XCO_2_ data are the total column through the atmosphere, extending above the troposphere. As the concentration of CO_2_ in the atmosphere is almost even in the vertical direction (Pearman and Garratt 1973; Woodwell et al. 1973), we used FTS SWIR L3 XCO_2_ data. The FTS SWIR Level 3 data extending from January to December 2010 were obtained from the GOSAT user interface gateway (https://data.gosat.nies.go.jp/GosatUserInterfaceGatway/guig/GuigPage/open.do).

### Tropopause height data

The tropopause is marked by large changes in the thermal, dynamical, and chemical structure of the atmosphere (Dentener et al. 2003; Dlugokencky et al. 2003; Bousquet et al. 2006). Various studies have attempted to elucidate the key factors that determine the latitude–altitude distribution of the tropopause (Haynes et al. 2001; Santer et al. 2003). Initial investigations were based on radiosonde data. More recently, analyses from numerical weather prediction centers and “reanalysis” products (Randel et al. 2000) have provided insights into the climatological properties of the tropopause, its seasonal cycle, and its secular variations on interannual and decadal timescales. Much of this work has been summarized by Seidel et al. (2001). These studies have revealed that the tropopause responds to a variety of influences, such as variations in solar radiation, atmospheric angular momentum, stratospheric ozone, and explosive volcanic eruptions (Randel et al. 2000).

We employed data from reanalyzes jointly obtained by the National Center for Environmental Prediction (NCEP) and the National Center for Atmospheric Research (NCAR) (Kalnay et al. 1996). In the internal metadata for the file, it specifies that the least significant digit is one to the right of the decimal point or tenth's place. The NCEP and NCAR Reanalysis Project began in 1991 as an outgrowth of the National Meteorological Center (NMC) Climate Data Assimilation System (CDAS) project. The project examined the apparent “climate changes” that resulted from many changes introduced in the NMC operational global data assimilation system (GDAS) over the last decade to improve the forecasts. Detailed descriptions of these data sets are given elsewhere (Pawson and Fiorino 1998). Here, it is sufficient to note that the operational models had different horizontal (T62 for NCEP) and vertical (17 pressure levels) resolutions. These 17 levels are 10, 20, 30, 50, 70, 100, 150, 200, 250, 300, 400, 500, 600, 700, 850, 925, and 1000 hPa. The NCEP data extend from January 1948 through to December 2011. We did not use NCEP temperatures prior to January 1979 due to well-documented problems of homogeneities that exist around the transition to satellite data assimilation (Santer et al. 1999).

**Figure 1:**
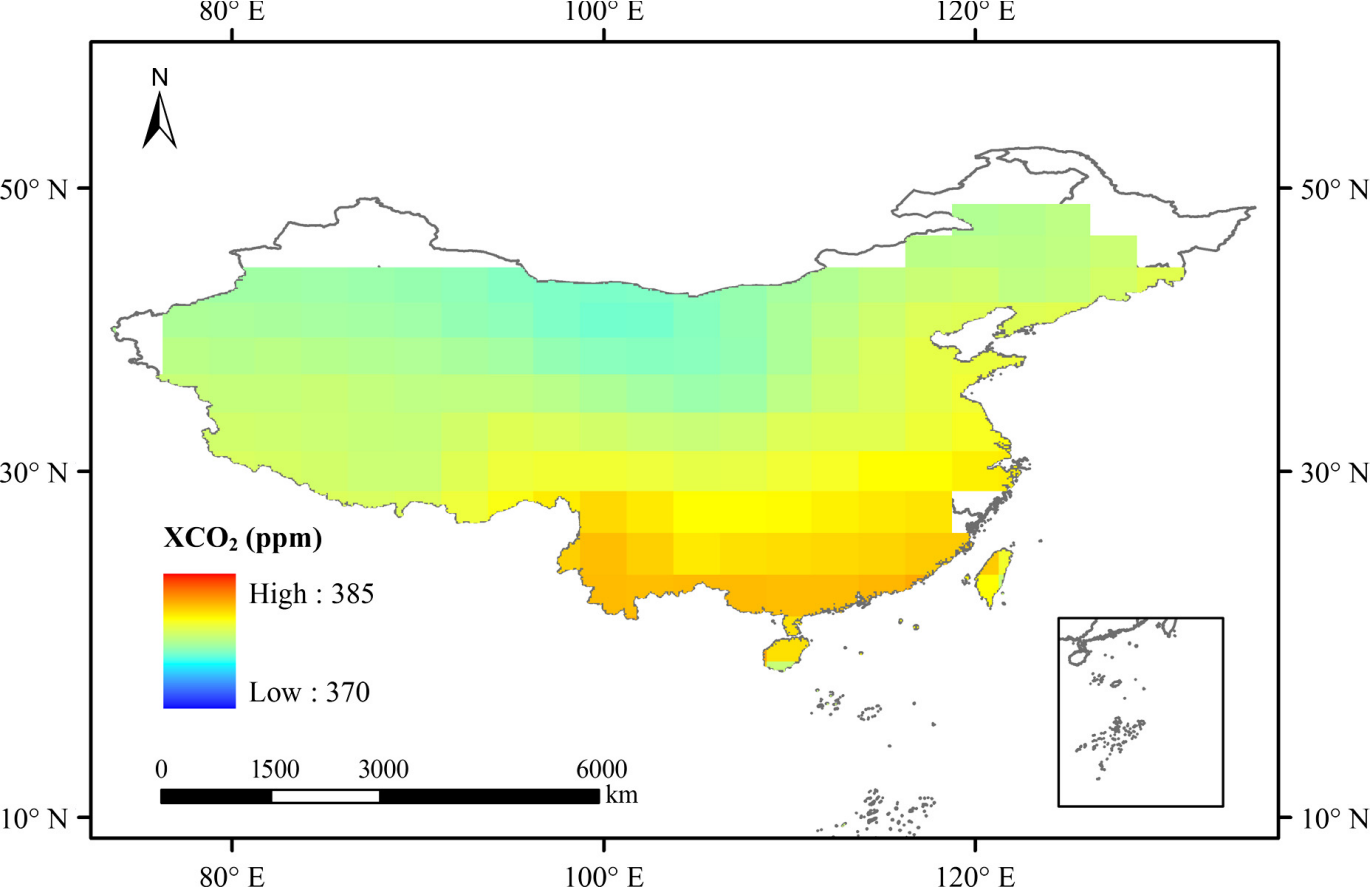
Distribution over China of the mean tropospheric CO_2_ concentration (XCO_2_) in January 2010 derived from GOSAT FTS SWIR Level 3 data.

In order to calculate tropospheric CO_2_ mass over a specified area, the concentration of CO_2_ and volumes of the troposphere in which it is contained are required. The FTS SWIR Level 3 data provide monthly average CO_2_ concentrations (XCO_2_), but there are missing values in some pixels (Fig. 1). In this study, grid cells lacking data were filled using kriging interpolation of data from the surrounding cells. From NCEP and NCAR reanalysis, tropopause pressure data were obtained, and from these values, tropopause height was calculated. As the terrain-following coordinate is used in tropopause pressure data, the altitude is not considered when calculating the tropopause height. According to the ground area and tropopause height, the volume of the troposphere was calculated. Then, the CO_2_ mass for the defined volume of troposphere was calculated_._

### Calculation of XCO_2_ by kriging interpolation

The FTS SWIR Level 3 data indicating CO_2_ concentration required georeferencing from the geographic lookup table (GLT) in ENVI. This is the premier software solution used by GIS, image analysts, scientists, and researchers for processing and analyzing geospatial imagery. Each image has 144 rows and 72 columns. Thus, 144 rows and 72 columns are required when using the “georeference from GLT” tool. The image was then clipped by China's administrative boundary in ENVI. To obtain the volume of each pixel, all images were transformed to equal-area projection. The Albers equal-area projection system, with the original longitude 105°E, a double standard parallel of 0°N and 0°N, the Beijing 1954 geodetic datum and the Krasovsky ellipsoid, was used.

**Figure 2:**
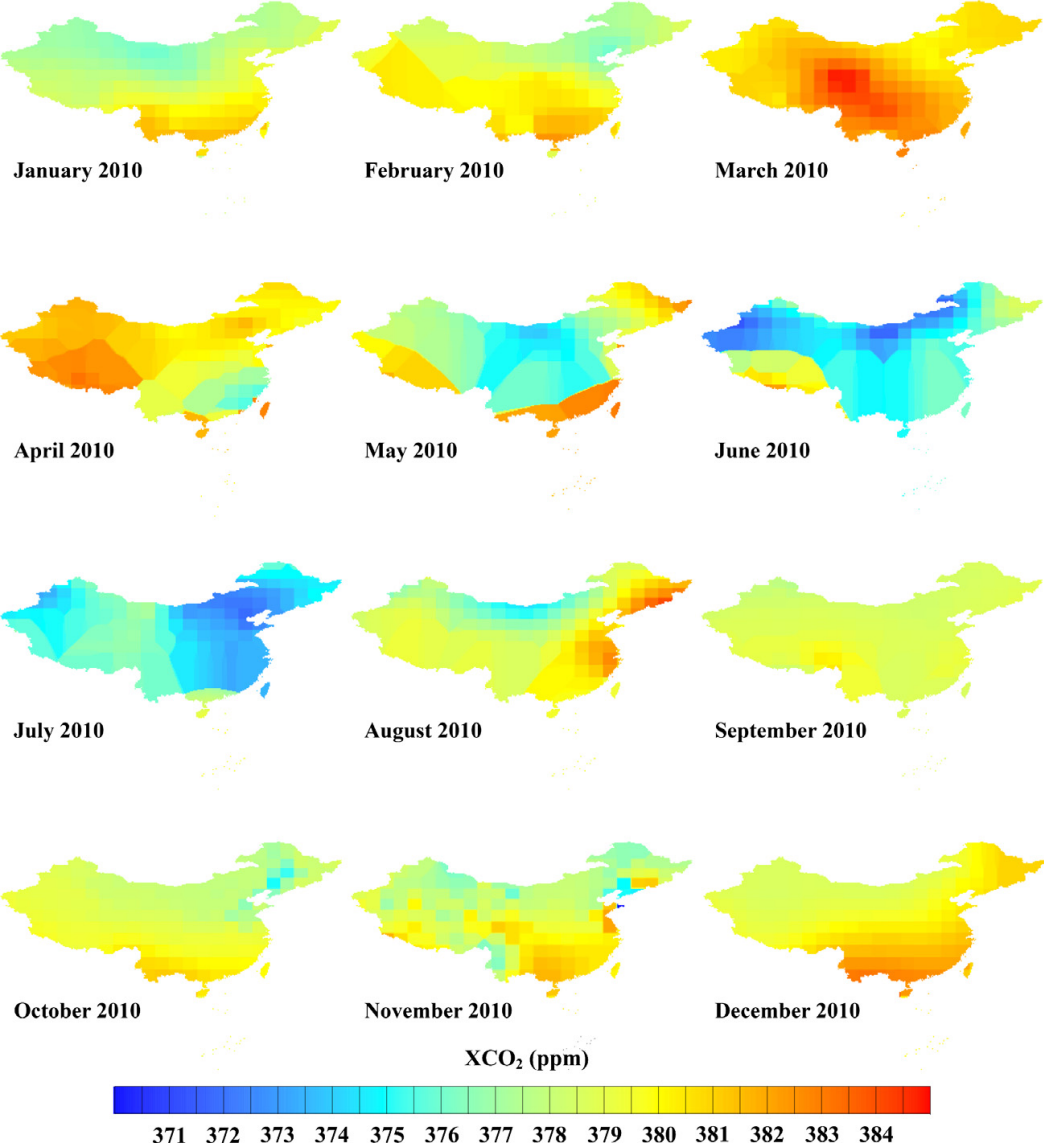
Distribution over China of monthly mean tropospheric CO_2_ concentrations (XCO_2_) during 2010 derived from FTS SWIR Level 3 data. Pixels without data were interpolated by kriging.

There were no data for parts of northern China (Fig. 1), so the pixels without data needed to be filled by interpolation. Interpolation methods commonly applied for estimating temperature or precipitation include distance weighting, polynomials interpolating, kriging, and splines (Nalder and Wein 1998). Distance weighting, which estimates the variable of interest by assigning more weight to closer points, is the simplest technique. Interpolating polynomials assigns a polynomial of an appropriate degree to the data points. Although higher degree polynomials provide a better fit, they may give totally unreasonable values between data points. Kriging, originally developed for mining ore estimation, assigns weights to minimize the variance and bias of the estimates. Spline methods, which are equivalent to kriging with a generalized covariance function, fit polynomials to a restricted set of points to provide a smooth, minimum curvature surface passing through the points(Lennon and Turner 1995). There is little evidence that any one method is optimal across a range of conditions; rather, it is important to determine the best method for each circumstance (Price et al., 2000). As kriging was used in processing FTS SWIR Level 2 data, the pixels that had no data in FTS SWIR Level 3 data were also interpolated by kriging in Figure 2.

The 2010 seasonal cycle of XCO_2_ over China is clearly shown with the lowest concentrations in summer and the highest in winter (Fig. 2). However, the XCO_2_ for March and April 2010 deviate from the seasonal cycle by being particularly high. Possibly, this is because there were fewer pixels with values in FTS SWIR Level 3 data for these months. Thus, values interpolated by kriging for these months may present larger patches that are not very accurate representations.

### Calculation of tropopause height

**Figure 3:**
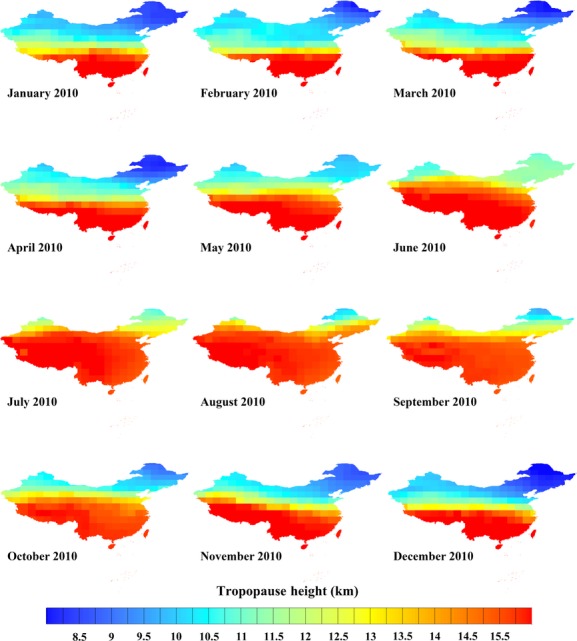
Monthly mean tropopause heights over China during 2010 derived from NCEP and NCAR reanalysis data.

The monthly mean tropopause was saved in the netCDF format. The Climate Data Operators (CDO) software is a collection of many operators for the standard processing of climate and numerical weather prediction (NWP) model outputs. The operators include simple statistical and arithmetic functions, data selection and subsampling tools, and spatial interpolation. The monthly mean troposphere pressures between January and December 2011 were selected from “pres.tropp.mon.mean.nc” by CDO 1.5.1. The monthly mean tropopause height was obtained by equation (1) (Filipiak 1999):


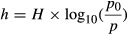
(1)

where *p* is the monthly mean troposphere pressure and *h* is the monthly mean tropopause height, *p*_0_ = 1000 hPa, *H* = 16 km. Distributions over China of mean tropopause heights for the months of 2010 are shown in Figure 3.

Monthly mean tropopause height showed a decreasing trend with increased latitude, varying between 15 km and 17 km in southern China, and between 8 km and 11 km in the north. Monthly mean tropopause height also varied with season being highest in summer and lowest in winter. Thus for most of China, the monthly mean tropopause height was higher than 15 km in July and was about 12 km in January.

### Calculation of the tropospheric CO_2_ mass over China

In order to ensure that the location and size of tropopause height and XCO_2_ estimates were the same, the monthly mean tropopause image and XCO_2_ image were resampled to 10 km pixel size, and then georeferenced in ArcGIS to make the two datasets align properly. The CO_2_ mass in the troposphere above a defined land area is equal to the volume of troposphere above the land area multiplied by the concentration of CO_2_ in that volume. Based on the area of each pixel and the tropopause height, the volume and mass of CO_2_ can be calculated. Details of the calculation processes are as follows:

The cell size of the raster data is 10 km, and therefore, the area of each pixel is 100 km^2^. The CO_2_ concentration in the atmosphere is measured in ppm. The ppm is adjusted to mg/m^3^ using equation (2):


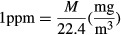
(2)

where *M* is the molecular weight with the molecular weight of CO_2_ equal to 44 and 22.4 is molar volume of the standard gas, so that


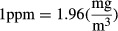
(3)

CO_2_ mass is calculated by equation (4):


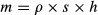
(4)

where *m* is the CO_2_ mass in the defined volume of troposphere, *ρ* is the concentration of CO_2,_ *s* is the area of each pixel and *h* is the tropopause height. Using the raster calculator in ArcGIS,


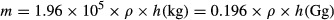
(5)

Gg, the abbreviation for gigagram is equal to 10^6^ kg.

The uncertainties were calculated as follows:


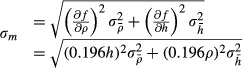
(6)

where *ρ* is the concentration of CO_2,_ *h* is the tropopause height, 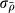 is the standard deviation of CO_2_ concentration, and 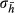 is the standard deviation of tropopause height.

### Comparison of tropospheric CO_2_ mass, ground emission data, population, and gross regional products

Ground estimates of CO_2_ emissions for the provinces based on the 2010 provincial energy statistics made by (Guan et al. 2012) are presented in Table 3 together with our estimates of tropospheric CO_2_ mass derived from satellite sensing. Populations and the gross regional products (GRP) of the provinces are also given in Table 3. Relationships between values in this Table were plotted, and their significance examined by regression analysis.

## Results and Discussion

### Spatial variation in tropospheric CO_2_ mass

**Figure 4:**
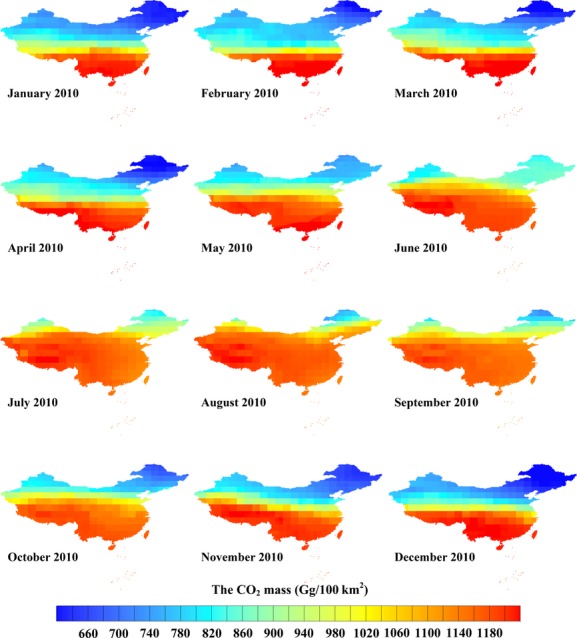
Distribution of monthly mean tropospheric CO_2_ mass per pixel (Gg/100 km^2^) over China during 2010 calculated from the volume of the troposphere and the concentration of CO_2_.

The monthly mean tropospheric CO_2_ mass calculated by equation (5) (Fig. 4) varied from 600 and 1250 Gg/100 km^2^ for different parts of China. Tropospheric CO_2_ mass was higher in southern than in northern China and higher in summer than in winter.

**Figure 5:**
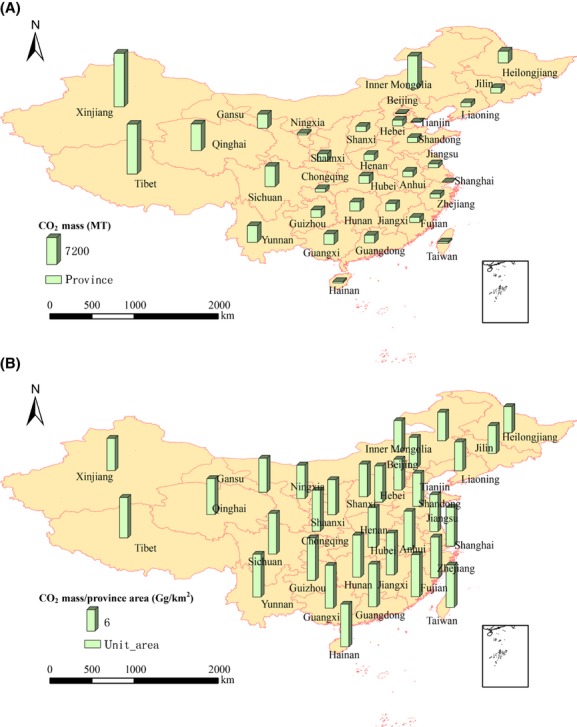
(A) Annual mean tropospheric CO_2_ mass (MT, million tonnes) for each province of China and (B) annual mean tropospheric CO_2_ mass per province area (Gg/km^2^) in 2010.

The spatial variation in annual mean tropospheric CO_2_ mass over China in 2010 obtained by the raster calculator in ArcGIS was calculated. From this calculation, using the zonal statistical function in ArcGIS, we calculated the annual mean tropospheric CO_2_ mass for each of the provincial areas of China (Table 1), and these masses are plotted for their geographical location on the map of China (Fig. 5A).

**Table 1:** The 2010 annual mean tropospheric CO_2_ masses and standard errors for the provincial areas of China

Province	Tropospheric CO_2_ mass (million tonnes)
Xinjiang	14,471 ± 63
Tibet	13,488 ± 67
Inner Mongolia	8881 ± 51
Qinghai	7230 ± 33
Sichuan	5523 ± 32
Yunnan	4535 ± 25
Gansu	3834 ± 21
Heilongjiang	3171 ± 20
Guangxi	2817 ± 16
Hunan	2448 ± 17
Guizhou	2087 ± 15
Guangdong	2077 ± 11
Hubei	2075 ± 14
Shaanxi	2018 ± 13
Jiangxi	1962 ± 13
Henan	1691 ± 11
Hebei	1600 ± 11
Jilin	1501 ± 9
Anhui	1496 ± 12
Shanxi	1427 ± 9
Fujian	1409 ± 8
Shandong	1346 ± 10
Liaoning	1160 ± 7
Zhejiang	1124 ± 7
Jiangsu	1046 ± 7
Chongqing	937 ± 6
Ningxia	466 ± 3
Taiwan	403 ± 2
Hainan	378 ± 1
Beijing	134 ± 1
Tianjin	99 ± 1
Shanghai	53 ± 1

Annual mean tropospheric CO_2_ masses for the provinces ranged from 53 ± 1 million tonnes (1 million tonnes = 10^9^ kg) to 14,470 ± 63 million tonnes. The five highest values were for Xinjiang, Tibet, Inner Mongolia, Qinghai, and Sichuan. The five lowest values were for the cities of Hong Kong, Shanghai, Tianjin, Beijing, and Hainan Island, all of which have small areas (Table 1, Fig. 5A). Thus, the obvious feature of provincial tropospheric CO_2_ masses is that their differences are primarily determined by provincial area. Consequently, tropospheric CO_2_ mass adjusted for provincial area has a different spatial pattern than the mean tropospheric CO_2_ masses of the provinces and is generally higher for eastern than for western provinces and higher for southern than for northern provinces (Fig. 5B). The north to south pattern is most likely a result of the increase in the height of the tropopause from north to south (Fig. 3), providing greater tropospheric volume to contain more CO_2_ mass (Fig. 4). The east to west pattern is not as readily explained, but a possibility is that the higher altitudes of the western provinces reduce the volume of troposphere containing CO_2_ mass.

### Temporal changes of monthly mean tropospheric CO_2_ mass

**Figure 6:**
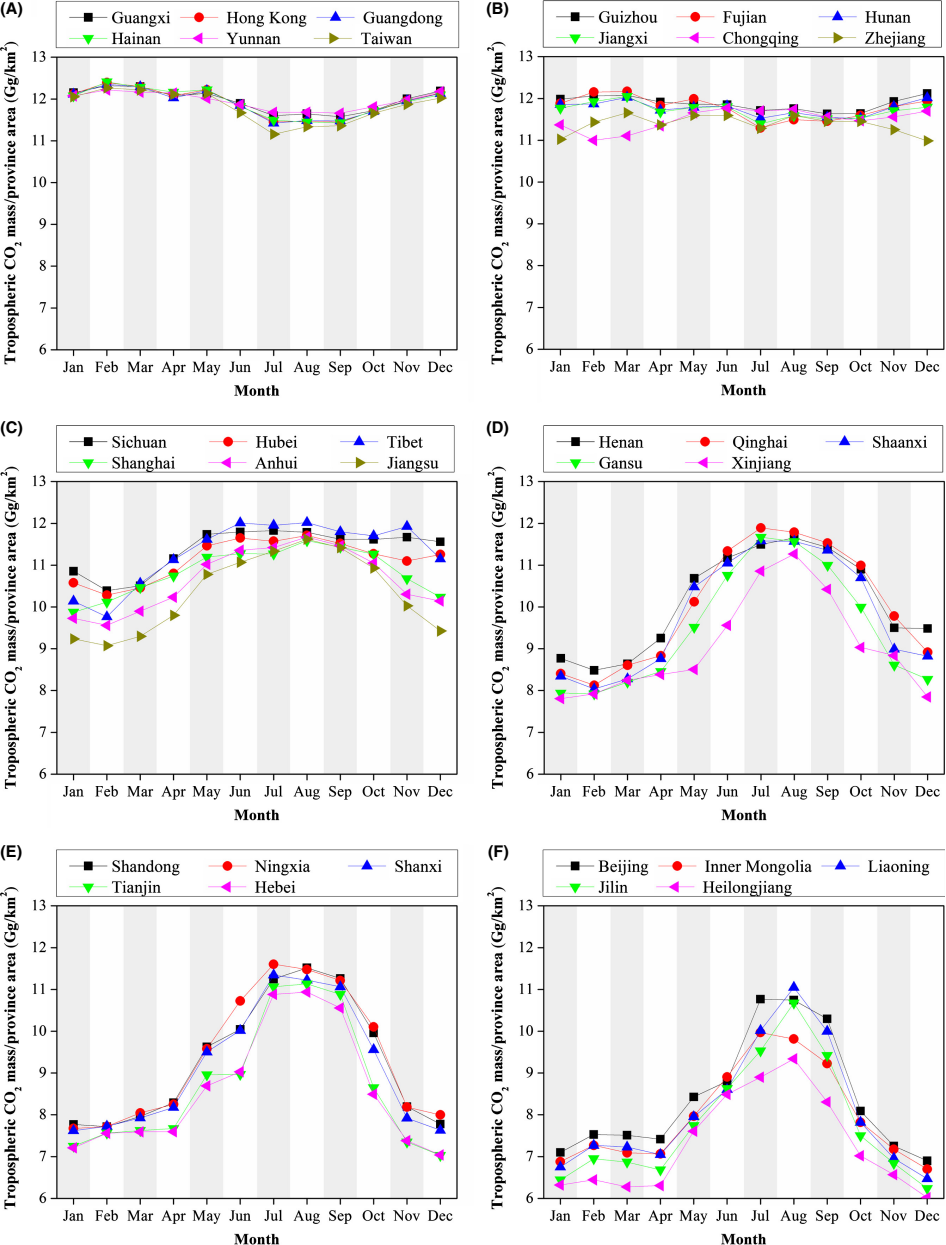
Temporal variation in monthly mean tropospheric CO_2_ mass/area for the provinces of China in 2010.

Monthly values of provincial tropospheric CO_2_ mass per provincial area between January and December 2010 were summarized by the function of “Zonal Statistics as a Table” in ArcGIS and are presented in Table 2 and plotted in Figure 6.

**Table 2:** 2010 monthly mean tropospheric CO_2_ masses / area and standard errors for the provinces of China

Province	January	February	March	April	May	June	July	August	September	October	November	December
Guangxi	12.15 ± 0.06	12.32 ± 0.06	12.30 ± 0.12	12.07 ± 0.05	12.14 ± 0.05	11.90 ± 0.10	11.60 ± 0.05	11.65 ± 0.06	11.58 ± 0.09	11.72 ± 0.06	12.00 ± 0.08	12.19 ± 0.08
Hong Kong	12.12 ± 0.06	12.39 ± 0.04	12.30 ± 0.03	12.08 ± 0.04	12.22 ± 0.05	11.84 ± 0.10	11.45 ± 0.08	11.47 ± 0.03	11.45 ± 0.09	11.72 ± 0.06	11.96 ± 0.08	12.15 ± 0.07
Guangdong	12.11 ± 0.07	12.35 ± 0.03	12.29 ± 0.07	12.03 ± 0.04	12.20 ± 0.04	11.84 ± 0.11	11.42 ± 0.12	11.48 ± 0.05	11.48 ± 0.10	11.70 ± 0.06	11.96 ± 0.10	12.13 ± 0.10
Hainan	12.08 ± 0.04	12.41 ± 0.04	12.28 ± 0.03	12.16 ± 0.03	12.22 ± 0.05	11.82 ± 0.10	11.50 ± 0.04	11.45 ± 0.03	11.43 ± 0.10	11.72 ± 0.05	11.93 ± 0.03	12.10 ± 0.04
Yunnan	12.07 ± 0.10	12.22 ± 0.06	12.16 ± 0.15	12.14 ± 0.05	12.02 ± 0.07	11.87 ± 0.07	11.68 ± 0.05	11.68 ± 0.05	11.66 ± 0.06	11.81 ± 0.09	11.97 ± 0.06	12.18 ± 0.04
Taiwan	12.05 ± 0.06	12.27 ± 0.03	12.22 ± 0.06	12.11 ± 0.04	12.13 ± 0.03	11.67 ± 0.23	11.16 ± 0.13	11.34 ± 0.07	11.36 ± 0.09	11.66 ± 0.06	11.87 ± 0.05	12.02 ± 0.11
Guizhou	11.98 ± 0.12	12.06 ± 0.06	12.08 ± 0.19	11.92 ± 0.05	11.86 ± 0.07	11.85 ± 0.10	11.71 ± 0.05	11.76 ± 0.10	11.63 ± 0.09	11.64 ± 0.07	11.93 ± 0.09	12.12 ± 0.05
Fujian	11.88 ± 0.07	12.15 ± 0.05	12.17 ± 0.05	11.83 ± 0.03	11.99 ± 0.03	11.77 ± 0.23	11.29 ± 0.13	11.49 ± 0.06	11.46 ± 0.10	11.59 ± 0.08	11.81 ± 0.05	11.94 ± 0.10
Hunan	11.86 ± 0.08	11.87 ± 0.06	12.03 ± 0.12	11.72 ± 0.05	11.79 ± 0.06	11.82 ± 0.10	11.53 ± 0.10	11.66 ± 0.09	11.55 ± 0.09	11.52 ± 0.07	11.80 ± 0.06	12.02 ± 0.08
Jiangxi	11.77 ± 0.08	11.93 ± 0.07	12.05 ± 0.05	11.68 ± 0.05	11.79 ± 0.05	11.80 ± 0.17	11.39 ± 0.13	11.59 ± 0.06	11.51 ± 0.10	11.53 ± 0.06	11.71 ± 0.05	11.79 ± 0.08
Chongqing	11.37 ± 0.08	11.00 ± 0.04	11.11 ± 0.10	11.35 ± 0.03	11.66 ± 0.11	11.77 ± 0.10	11.70 ± 0.05	11.74 ± 0.10	11.55 ± 0.09	11.47 ± 0.06	11.56 ± 0.09	11.70 ± 0.06
Zhejiang	11.02 ± 0.05	11.44 ± 0.06	11.66 ± 0.05	11.37 ± 0.07	11.60 ± 0.07	11.59 ± 0.23	11.29 ± 0.13	11.59 ± 0.06	11.45 ± 0.09	11.45 ± 0.08	11.25 ± 0.05	10.99 ± 0.09
Sichuan	10.86 ± 0.08	10.39 ± 0.05	10.51 ± 0.08	11.15 ± 0.03	11.74 ± 0.10	11.79 ± 0.07	11.83 ± 0.06	11.79 ± 0.06	11.62 ± 0.06	11.62 ± 0.05	11.67 ± 0.06	11.56 ± 0.08
Hubei	10.58 ± 0.05	10.28 ± 0.06	10.46 ± 0.05	10.80 ± 0.07	11.46 ± 0.08	11.65 ± 0.11	11.57 ± 0.10	11.71 ± 0.10	11.52 ± 0.09	11.27 ± 0.07	11.10 ± 0.06	11.26 ± 0.06
Tibet	10.14 ± 0.08	9.76 ± 0.05	10.57 ± 0.07	11.13 ± 0.04	11.62 ± 0.03	12.01 ± 0.05	11.96 ± 0.04	12.02 ± 0.08	11.80 ± 0.09	11.70 ± 0.05	11.93 ± 0.07	11.15 ± 0.06
Shanghai	9.88 ± 0.04	10.12 ± 0.03	10.46 ± 0.05	10.74 ± 0.08	11.19 ± 0.07	11.27 ± 0.22	11.27 ± 0.13	11.59 ± 0.06	11.43 ± 0.08	11.26 ± 0.08	10.68 ± 0.04	10.24 ± 0.06
Anhui	9.73 ± 0.04	9.56 ± 0.04	9.90 ± 0.04	10.23 ± 0.07	11.02 ± 0.07	11.36 ± 0.22	11.42 ± 0.13	11.67 ± 0.08	11.47 ± 0.09	11.05 ± 0.07	10.31 ± 0.06	10.14 ± 0.06
Jiangsu	9.24 ± 0.04	9.07 ± 0.03	9.30 ± 0.04	9.80 ± 0.07	10.78 ± 0.07	11.07 ± 0.22	11.34 ± 0.13	11.62 ± 0.06	11.42 ± 0.08	10.93 ± 0.08	10.03 ± 0.11	9.43 ± 0.06
Henan	8.77 ± 0.06	8.48 ± 0.04	8.64 ± 0.04	9.26 ± 0.06	10.69 ± 0.07	11.17 ± 0.10	11.50 ± 0.13	11.65 ± 0.10	11.43 ± 0.08	10.90 ± 0.07	9.50 ± 0.05	9.48 ± 0.03
Qinghai	8.41 ± 0.05	8.13 ± 0.04	8.61 ± 0.03	8.83 ± 0.03	10.12 ± 0.04	11.34 ± 0.04	11.89 ± 0.05	11.79 ± 0.08	11.53 ± 0.05	10.99 ± 0.05	9.78 ± 0.05	8.92 ± 0.04
Shaanxi	8.34 ± 0.05	8.04 ± 0.06	8.28 ± 0.02	8.76 ± 0.02	10.48 ± 0.09	11.05 ± 0.09	11.58 ± 0.05	11.57 ± 0.10	11.36 ± 0.07	10.70 ± 0.08	8.99 ± 0.06	8.82 ± 0.04
Gansu	7.94 ± 0.05	7.92 ± 0.04	8.20 ± 0.03	8.46 ± 0.02	9.52 ± 0.07	10.76 ± 0.06	11.67 ± 0.10	11.57 ± 0.08	11.00 ± 0.05	9.99 ± 0.05	8.61 ± 0.04	8.27 ± 0.03
Xinjiang	7.81 ± 0.03	7.92 ± 0.03	8.25 ± 0.03	8.38 ± 0.04	8.50 ± 0.05	9.56 ± 0.07	10.86 ± 0.04	11.27 ± 0.05	10.42 ± 0.05	9.03 ± 0.03	8.84 ± 0.04	7.85 ± 0.05
Shandong	7.77 ± 0.08	7.72 ± 0.02	7.96 ± 0.03	8.29 ± 0.06	9.63 ± 0.06	10.04 ± 0.20	11.25 ± 0.13	11.52 ± 0.07	11.26 ± 0.08	9.96 ± 0.07	8.20 ± 0.07	7.78 ± 0.02
Ningxia	7.67 ± 0.08	7.72 ± 0.06	8.05 ± 0.01	8.25 ± 0.02	9.58 ± 0.09	10.72 ± 0.09	11.60 ± 0.05	11.48 ± 0.11	11.21 ± 0.06	10.10 ± 0.05	8.18 ± 0.04	8.00 ± 0.03
Shanxi	7.62 ± 0.04	7.73 ± 0.07	7.93 ± 0.04	8.18 ± 0.05	9.50 ± 0.06	10.02 ± 0.06	11.34 ± 0.011	11.22 ± 0.06	11.06 ± 0.05	9.56 ± 0.07	7.92 ± 0.03	7.63 ± 0.05
Tianjin	7.25 ± 0.09	7.57 ± 0.03	7.63 ± 0.02	7.67 ± 0.05	8.96 ± 0.06	8.97 ± 0.18	11.06 ± 0.13	11.13 ± 0.02	10.88 ± 0.06	8.65 ± 0.13	7.35 ± 0.03	7.03 ± 0.02
Hebei	7.21 ± 0.07	7.56 ± 0.02	7.59 ± 0.03	7.60 ± 0.05	8.69 ± 0.06	9.03 ± 0.14	10.88 ± 0.12	10.94 ± 0.03	10.56 ± 0.05	8.49 ± 0.07	7.38 ± 0.05	7.04 ± 0.02
Beijing	7.10 ± 0.09	7.53 ± 0.02	7.51 ± 0.02	7.42 ± 0.05	8.43 ± 0.05	8.80 ± 0.17	10.77 ± 0.12	10.74 ± 0.02	10.30 ± 0.05	8.09 ± 0.09	7.25 ± 0.04	6.90 ± 0.01
Inner Mongolia	6.88 ± 0.03	7.27 ± 0.04	7.09 ± 0.02	7.07 ± 0.02	7.97 ± 0.07	8.91 ± 0.11	9.97 ± 0.06	9.81 ± 0.04	9.23 ± 0.04	7.82 ± 0.05	7.18 ± 0.03	6.70 ± 0.03
Liaoning	6.75 ± 0.02	7.27 ± 0.04	7.23 ± 0.03	7.05 ± 0.03	7.95 ± 0.13	8.60 ± 0.17	10.02 ± 0.11	11.05 ± 0.02	10.00 ± 0.07	7.82 ± 0.07	6.96 ± 0.03	6.47 ± 0.02
Jilin	6.44 ± 0.03	6.95 ± 0.04	6.87 ± 0.03	6.68 ± 0.01	7.74 ± 0.11	8.63 ± 0.14	9.53 ± 0.07	10.68 ± 0.05	9.42 ± 0.05	7.50 ± 0.06	6.84 ± 0.03	6.24 ± 0.02
Heilongjiang	6.32 ± 0.03	6.45 ± 0.03	6.28 ± 0.02	6.31 ± 0.01	7.61 ± 0.05	8.49 ± 0.12	8.90 ± 0.08	9.34 ± 0.07	8.31 ± 0.06	7.02 ± 0.06	6.57 ± 0.02	6.02 ± 0.02

The temporal monthly variation in tropospheric CO_2_ mass per provincial area over China in 2010 ranged from 6 to 13 Gg/km^2^. For most provinces (Fig. 6C–F), the tropospheric CO_2_ mass per square kilometers varied in an annual cycle being highest in July and August and lowest in January and February. Monthly changes of tropospheric CO_2_ mass for Guangxi, Hong Kong, Guangdong, Hainan, Yunnan, Taiwan, Guizhou, and Fujian were between 11 and 12.5 Gg/km^2^ (Fig. 6A,B).

Tropopause height, which is highest in summer and lowest in winter, a pattern that is stronger as latitude increases (Fig. 3), appears to be the most important factor affecting seasonal variation in tropospheric CO_2_ mass per area for China. Other factors may contribute to this pattern including seasonal variation in carbon fixation by photosynthesis emissions from decomposition of vegetation residues, seasonal variation in burning carbon for domestic heating, and industrial emissions.

### Comparison of CO_2_ emission estimates with tropospheric CO_2_ mass estimates

**Figure 7:**
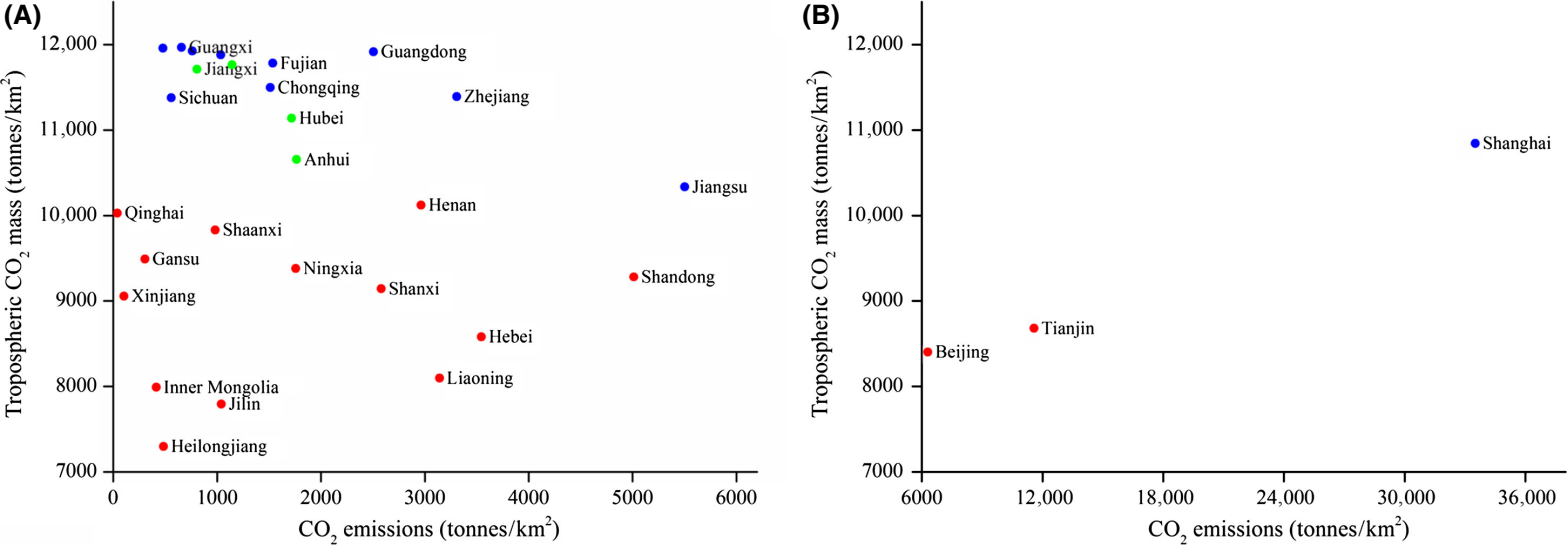
Scatter plot of provincial CO_2_ emissions per unit area based on 2010 provincial energy statistics against remotely sensed tropospheric CO_2_ mass per unit area for the provinces of China. Red, blue, and green circles denote that these provinces located in the south, north, and centre of China, respectively. (A) Provinces excluding Shanghai, Beijing, and Tianjin. (B) Shanghai, Beijing, and Tianjin.

Guan et al. (2012) obtained publicly available energy statistics from Chinese authorities and followed the Intergovernment Panel on Climate Change (IPCC) emission accounting approach to compile an emission inventory for every Chinese province. Our estimations of the tropospheric CO_2_ mass and the emission estimates based on the 2010 provincial energy statistics made by Guan et al. (2012) are presented in Table 3 and are plotted with reference to each other in Figure 7.

**Table 3:** 2010 provincial gross regional products (GRP), populations, CO_2_ emissions based on statistical data per square kilometer (Guan et al. 2012), remotely sensed tropospheric CO_2_ mass per square kilometer, and the percentage proportion of tropospheric CO_2_ provided by current annual emissions. Provinces are listed in the order from highest to lowest% of current annual emission to remotely sensed tropospheric CO_2_ mass per unit area

Province	Population (10 thousand)	Gross Regional Product (100 million yuan)	CO_2_ emissions based on 2010 provincial energy statistics (million tonnes)	Remotely Sensed tropospheric CO_2_ mass (million tonnes)	CO_2_ emissions based on 2010 provincial energy statistics per square kilometer (tonnes/km^2^)	Remotely Sensed tropospheric CO_2_ per square kilometer (tonnes/km^2^)	Percentage of CO_2_ emissions to tropospheric CO_2_ mass (%)
Shanghai	2301.91	17165.98	211.26	53.14	33501.43	10844.35	308.93
Tianjin	1293.82	9224.46	134.36	98.95	11576.77	8679.39	133.38
Beijing	1961.24	14113.60	103.05	134.46	6295.05	8403.63	74.91
Shandong	9579.31	39169.92	769.12	1345.72	5013.10	9280.83	54.02
Jiangsu	7865.99	41425.48	555.56	1045.88	5503.21	10334.78	53.25
Hebei	7185.42	20394.26	663.18	1600.39	3545.56	8581.18	41.32
Liaoning	4374.63	18457.30	456.38	1159.55	3141.81	8097.42	38.80
Henan	9402.36	23092.36	490.92	1691.42	2964.49	10122.20	29.29
Zhejiang	5442.69	27722.31	337.48	1124.33	3307.17	11391.39	29.03
Shanxi	3571.21	9200.86	403.45	1427.17	2579.92	9142.66	28.22
Guangdong	10430.31	46013.06	443.59	2077.04	2504.97	11916.47	21.02
Ningxia	630.14	1689.65	91.11	466.25	1757.08	9381.33	18.73
Anhui	5950.05	12359.33	247.75	1495.96	1764.64	10654.99	16.56
Hubei	5723.77	15967.61	319.61	2075.15	1716.83	11138.75	15.41
Jilin	2746.23	8667.58	198.36	1501.27	1041.04	7794.76	13.36
Chongqing	2884.62	7925.58	124.86	937.03	1512.74	11497.30	13.16
Fujian	3689.42	14737.12	187.30	1409.13	1536.58	11782.02	13.04
Shaanxi	3732.74	10123.48	202.27	2018.34	982.37	9831.17	9.99
Hunan	6568.37	16037.96	243.02	2448.11	1144.07	11764.10	9.73
Guizhou	3474.65	4602.16	182.36	2087.11	1034.65	11878.83	8.71
Jiangxi	4456.75	9451.26	134.84	1961.70	805.97	11711.64	6.88
Heilongjiang	3831.22	10368.60	217.38	3171.16	482.99	7300.09	6.62
Hainan	867.15	2064.50	25.82	378.03	759.88	11925.11	6.37
Guangxi	4602.66	9569.85	155.79	2817.20	657.87	11967.71	5.50
Inner Mongolia	2470.63	11672.00	474.35	8881.04	414.22	7990.86	5.18
Sichuan	8041.82	17185.48	270.10	5523.28	557.70	11376.48	4.90
Yunnan	4596.62	7224.18	183.64	4534.91	478.26	11956.00	4.00
Gansu	2557.53	4120.75	123.44	3833.85	304.82	9492.08	3.21
Xinjiang	2181.33	5437.47	166.75	14470.70	102.10	9057.21	1.13
Qinghai	562.67	1350.43	28.88	7229.56	40.36	10028.52	0.40
Total			8145.98	78997.82			10.31

Comparison of the tropospheric CO_2_ mass and emission estimates (Fig. 7A) indicates that they are not significantly related (*r* = 0.24). The main pattern indicated is for troposphere CO_2_ mass to decrease from southern to northern provinces (y axis).

The mean percentage of total annual process CO_2_ emissions averaged for all the provinces of China relative to the total tropospheric CO_2_ mass based on our remote sensing data estimates is 10.3% (Table 3). This percentage differs widely between provinces varying from 308.9% for Shanghai to 0.40% for Qinghai. The percentage of emissions relative to the estimated tropospheric CO_2_ mass over the area of provinces is higher than 20% for Shanghai, Tianjin, Beijing, Shandong, Jiangsu, Hebei, Liaoning, Zhejiang, Henan, Shanxi, and Guangdong. The three provincial-level municipalities, Beijing, Tianjin, and Shanghai, with exceptionally high proportions of emissions compared with the tropospheric CO_2_ mass above their areas, have developed economies intensified on much smaller areas leading to higher CO_2_ emissions per unit area. Jiangsu, Zhejiang, Shandong, and Guangdong are more developed provinces on the eastern coast of China, and their CO_2_ emissions are higher than for the remaining provinces, Qinghai, Xinjiang, Gansu, Yunnan, Sichuan, Inner Mongolia, Guangxi, Heilongjiang, Hainan, Jiangxi, and Guizhou. For these provinces, the proportion of CO_2_ emission relative to tropospheric CO_2_ mass is less than 10%. They are located in west, north, and central China and do not have well-developed industrial economies, and consequently, their unit area CO_2_ emissions are lower.

### Relationships of population and GRP with CO_2_ emissions

To consider effects of economic development and population numbers on CO_2_ emissions, we compared CO_2_ emissions based on 2010 provincial energy statistics with GRP and population (Table 3). The GRP data were obtained from 3 to 2 gross regional products (2010) in 2011 China Statistical Yearbook for Regional Economy, and the population data were obtained from 3 to 7 region's population and sex ratio in 2011 China Statistical Yearbook. As we had no GRP data for Tibet, Hong Kong, Macao, and Taiwan, they are not included in the analyses.

**Figure 8:**
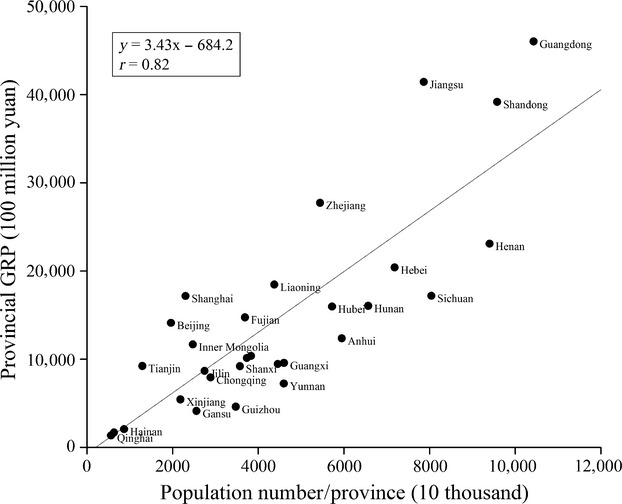
Relationship between Chinese provincial populations and provincial GRP.

**Figure 9:**
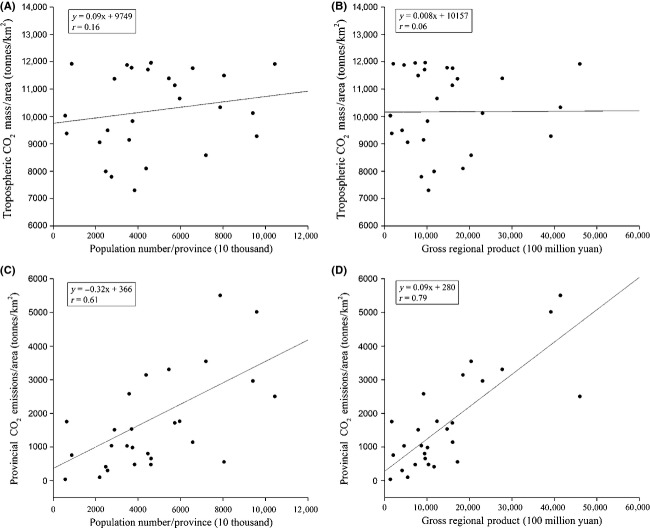
(A) Relationship between provincial unit area troposphere CO_2_ mass and population numbers of the provinces of China. (B) Relationship between provincial unit area unit area troposphere CO_2_ mass and GRP for the provinces of China. (C) Relationship between unit area CO_2_ emissions and population numbers of the provinces of China. (D) Relationship between provincial unit area CO_2_ emissions and GRP for the provinces of China. The three provincial municipalities, Shanghai, Tianjin, and Beijing, are not included in the four figures.

**Figure 10:**
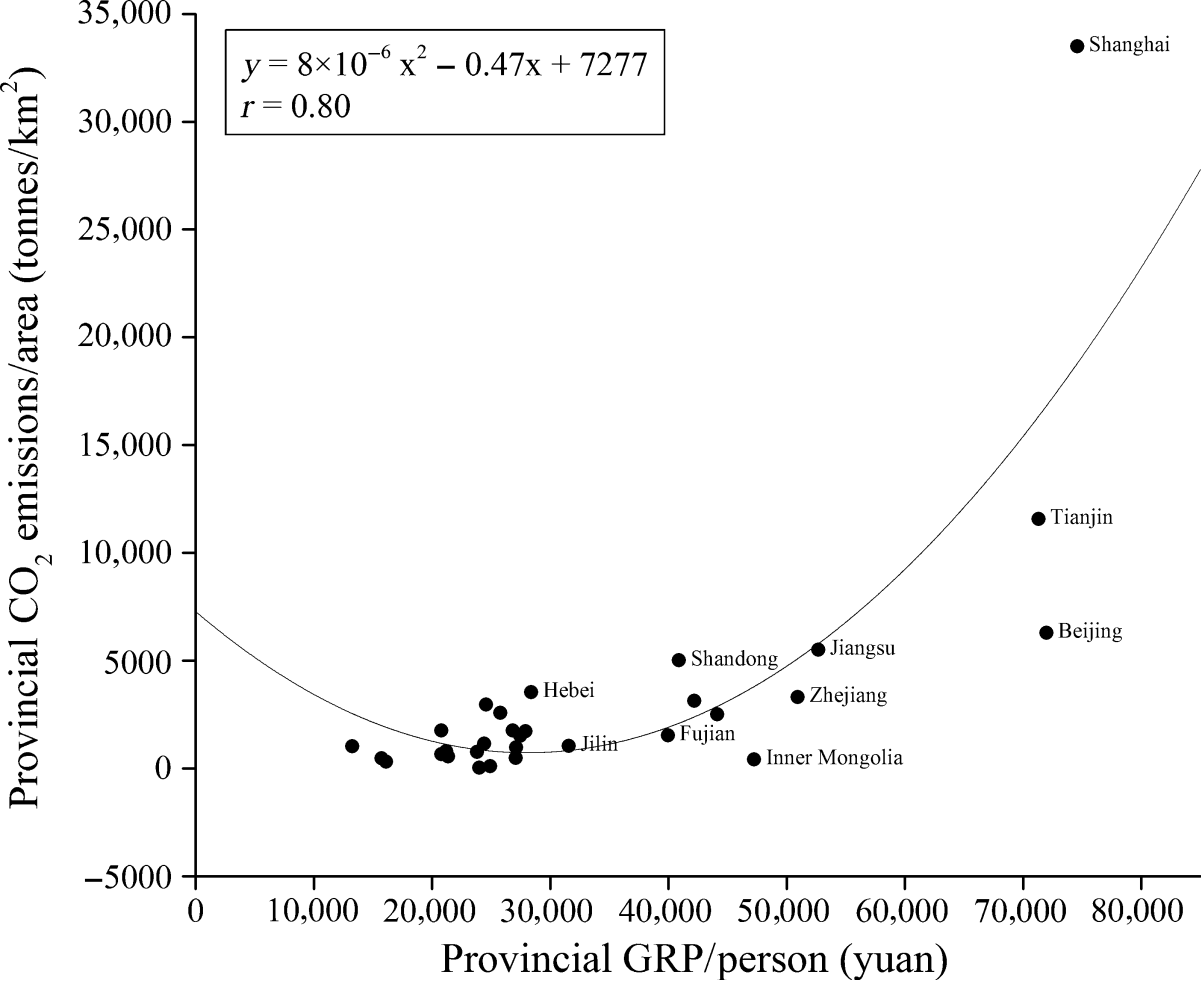
Relationship between CO_2_ emissions and GRP per person for the provinces of China.

The relationship between provincial population numbers and GRP is highly significant (*r* = 0.82, *P* = 0.03) (Fig. 8). The regression separates provinces with relatively high GRP for their populations, which are above the regression line, from those with low GRP for their populations, which are below the line. The relationships between provincial tropospheric CO_2_ mass and populations and GRP were not significant (Fig. 9A,B). Considered separately, the relationships between provincial populations and GRP and CO_2_ emissions were not significant, but removal of the three provincial municipalities, Shanghai, Tianjin, and Beijing, indicated positive relationships between provincial populations and GRP and CO_2_ emissions (Fig. 9C,D). When considered as provincial GRP/person, the regression is significant as a quadratic relationship (Fig. 10). In particular, this relationship indicates the very high level of CO_2_ emissions from Shanghai, and to a lesser extent by Tianjin and Beijing, compared with the other provinces. It is suggested that the high CO_2_ emissions from these municipalities are rapidly dispersed into the wider volume of the troposphere over China so as to be not detected as variations in CO_2_ mass in the volumes of troposphere above them.

## Conclusions

Our determinations of tropospheric CO_2_ mass for the provincial areas of China have not shown relationships between human population density and economic activity in the way indicated with ground-based estimates of CO_2_ emissions. There are several possible explanations for this. First, CO_2_ emissions are quickly mixed and dispersed in the atmosphere (Foucher et al. 2011). Atmospheric circulation transports CO_2_ from surface to very broad volumes of troposphere both laterally and vertically (Keppel-Aleks et al. 2011). Second, an important consideration is which layers of the atmosphere were sensed for CO_2_ concentration by GOSAT. If lower altitude layers were specifically sensed, then it is more likely that they would show a relationship to ground emissions. However, the FTS SWIR Level 3 data that were available for our calculations of CO_2_ mass were column-averaged mixing ratios of CO_2_ over the broader depth of the atmosphere and not just the troposphere. Third, the adjustment for troposphere height may incorrectly distort the estimates of CO_2_ mass. Altitude of the land surface may also have a significant effect on troposphere CO_2_ mass.

In addition to the anthropogenic emissions into the troposphere that we have specifically considered, the column CO_2_ mass in the bulk atmosphere has many other sources. Besides the broad spatial patterns of tropospheric CO_2_ mass related to seasonal cycles and latitude (Fig. 3), there are indications of longitudinal variations that may indicate other influences on tropospheric CO_2_ mass, that is, distance from coastal-oceanic influences. Monitoring for several years is required to confirm the existence of such effects. Consequently, we plan to further quantify variations of spatial and temporal variations of tropospheric CO_2_ mass over China in order to obtain better understanding of the causes and mechanisms of these variations.
